# Dobutamine-Induced Takotsubo Cardiomyopathy With Severe Left Ventricular Dysfunction

**DOI:** 10.7759/cureus.50390

**Published:** 2023-12-12

**Authors:** Safa Fatima, Zain S Mohiuddin, Michael D Bage, Lidiya Sul

**Affiliations:** 1 Internal Medicine, Mercy Health System, Javon Bea Hospital, Rockford, USA; 2 Internal Medicine, West Virginia School of Osteopathic Medicine, Princeton, USA; 3 Internal Medicine, Western Reserve Hospital, Cuyahoga Falls, USA; 4 Cardiology, Western Reserve Hospital, Cuyahoga Falls, USA; 5 Internal Medicine, Cleveland Clinic, Cleveland, USA

**Keywords:** takotsubo cardiomyopathy, acute coronary syndrome, unusual causes of persistent chest pain, dopamine, left ventricular systolic dysfunction

## Abstract

Takotsubo cardiomyopathy (TCM) is characterized by severe left ventricular dysfunction. It presents as an acute coronary syndrome; however, the difference lies in the lack of coronary artery obstruction during a coronary angiogram. The left ventricular dysfunction extends beyond the area supplied with concordant coronary arteries. We describe a case of a 41-year-old female evaluated for acute coronary syndrome, later diagnosed with a unique reverse subtype of TCM.

## Introduction

Takotsubo cardiomyopathy (TCM) is characterized by left-ventricular systolic dysfunction. Pathogenesis is still yet to be clearly defined; however, TCM is likely the end result of a catecholamine surge, systemic inflammation, endothelial dysfunction, coronary vasospasm, and excessive activation of the sympathetic nervous system. Dobutamine stress testing mimics the effects of this endogenous catecholamine storm that is produced when someone is under stress. Under stressed conditions, patients with TCM present with precordial chest pain, ECG changes, and increased cardiac biomarkers, mimicking acute coronary syndrome (ACS). Unlike myocardial infarction, cardiac catheterization shows unobstructed coronary arteries and ballooning of the cardiac apex, forming the classic “ceramic pot” appearance on imaging [1]. Here, we describe a case of TCM presenting with atypical findings of mid-ventricular wall hypokinesis with basilar and apical wall sparing, which is different from prototypical findings.

## Case presentation

A 41-year-old female with a history of essential hypertension on multiple anti-hypertensives, a recent lower extremity fracture presented after a mechanical fall. Her family history was significant for premature coronary disease in her father, who had his first myocardial infarction at 41 years of age. She had six children and had no postpartum complications. She had a 25-pack-year smoking history and presented with typical substernal chest pain ongoing for three to four days, getting worse with exertion and alleviated with rest. ECG demonstrated sinus rhythm and nonspecific anterior T-wave inversion in V1. High-sensitivity troponin T levels were not elevated. A chest radiograph showed no acute cardiopulmonary process. Blood counts, electrolytes, renal function, and liver function tests were unremarkable. A dobutamine stress echocardiogram was performed.

A dobutamine stress echo using a standard dobutamine/atropine protocol was used. Dobutamine was infused in incremental doses, starting at 10 mcg/kg/min doses at three-minute intervals, to a maximum dose of 40 mcg/kg/min. A total of 0.75 mg of atropine was administered at a high dose of dobutamine to reach a diagnostic heart rate. Left ventricular systolic function was normal, and left ventricular ejection fraction (LVEF) was estimated to be 73% by the modified Simpson method. The LVEF and wall motion were normal at rest.

The maximum heart rate achieved was 98% of the maximum age-predicted heart rate. The patient developed crushing substernal chest pain along with 1.5 to 2 mm of down-sloping ST depression in the inferolateral leads. The left ventricle failed to augment as expected with a high dose of dobutamine, and there was superimposed regional variability. The lateral and anterior walls became hypokinetic. This was an abnormal finding suggestive of possible multi-vessel coronary artery disease. The patient was taken for an emergent cardiac catheterization.

**Figure 1:**
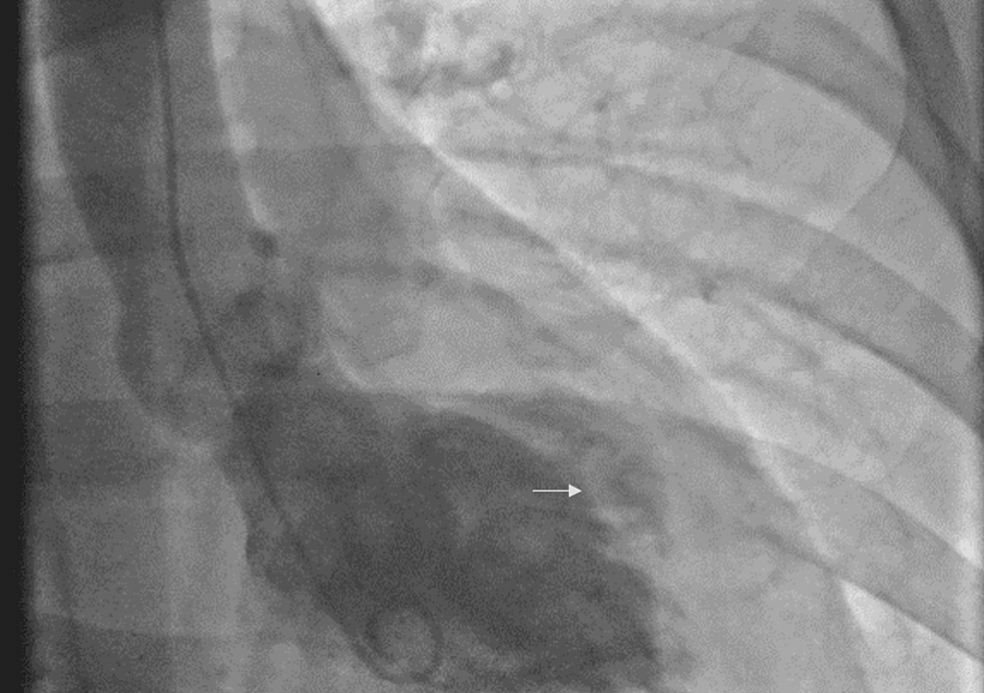
Ventriculogram showing mid-ventricular dysfunction due to reverse subtype of TCM The ventriculogram shows mid-ventricular ballooning as seen in the reverse subtype of TCM

During the cardiac catheterization, she was found to have normal coronary arteries with no evidence of calcification or irregularity of the vessels. The left ventriculogram (Figure 1) demonstrated a pattern of reverse TCM with akinesis of the diaphragmatic segment of the left ventricle and dyskinesis of the high anterolateral wall with preserved apical and basal contraction. The serum troponins were mildly elevated. The patient was informed of the normal coronary arteries and the features of TCM. She was commenced on beta-blockade and was asked to follow up with her primary cardiologist.

## Discussion

**Figure 2:**
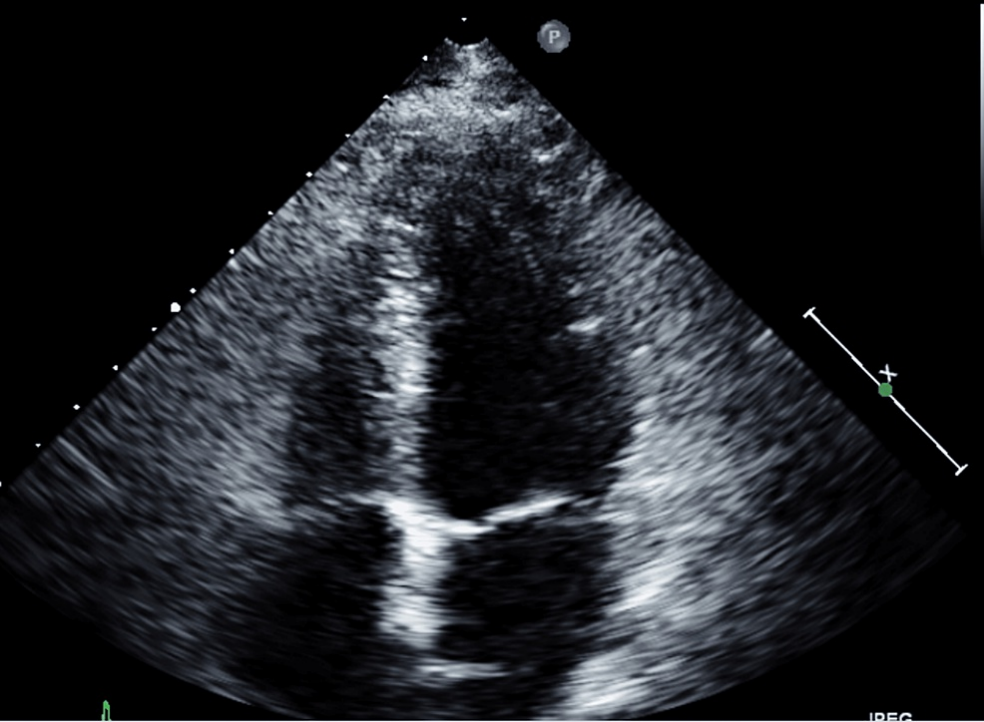
Normal chambered heart

Currently, the prevalence of TCM is 5.2/100,000 for females and 0.6/100,000 for males. Several etiological factors have been reported, but the most documented and reported findings show there is a predisposition for post-menopausal women undergoing severe emotional, mental, or physical stress inducing a catecholamine surge. Studies have demonstrated that emotional stress is strongly related to the development of TCM in about 20-39% of cases [1,2]. One of the most popular theories is the activation of the sympathetic nervous system in response to stress, which results in a massive release of catecholamines [3-5]. Due to this apical dysfunction, myocardial stunning and paradoxical vasodilation develop, causing a decrease in cardiac output, systemic hypotension, and acute heart failure. These effects are a cardioprotective mechanism. The apex of the heart has a greater response to adrenergic stimulation than the base, which is why imaging studies of patients with TCM often show apical hypokinesis and ballooning. Less commonly, inverted and mid-ventricular dysfunction are also found; these features are significantly different from the typical four-chambered cardiac structure (Figure 2).

**Figure 3:**
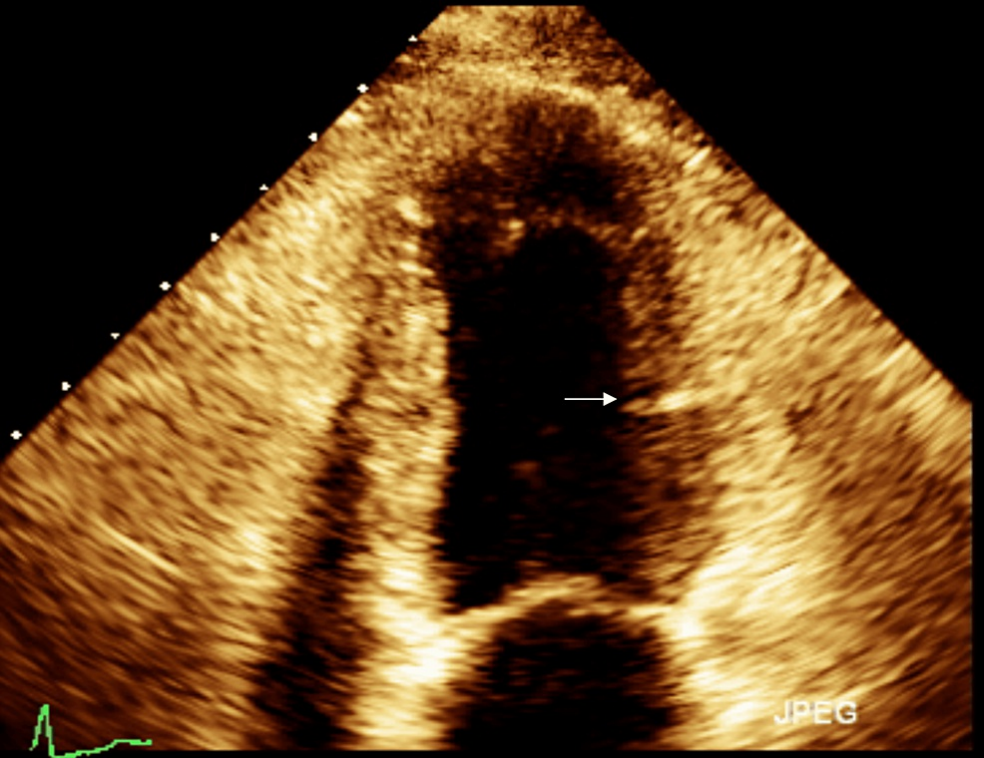
The ceramic pot appearance: a reverse subtype of TCM Akinesis of a diaphragmatic segment of the left ventricle (white arrow) with preserved apical and basal contraction of the mid-ventricular segment causing a ceramic pot appearance to be seen

In our patient, there was an evident history of acute stressors in her life. She presented with typical symptoms, ECG changes, and dobutamine stress test findings suggesting coronary artery disease. Cardiac catheterization ruled out ACS but revealed evidence of TCM. A diagnosis of TCM was confirmed, but it did not demonstrate the typical apical ballooning pattern characteristic of TCM. Instead, we found the reverse type of TCM with wall motion abnormalities in the high anterolateral and diaphragmatic segments of the left ventricle (Figure 3). Although poor outcomes have not been reported often with TCM, it is extremely important to diagnose patients accurately to appropriately offer prognostication. Studies are needed to determine if medications used in heart failure provide mortality benefits.

## Conclusions

Pharmacological stress testing, specifically with dobutamine, has been used in clinical practice for several years to evaluate the ischemic effects of coronary artery disease. TCM, although rare, must be considered in the differential diagnosis for any patient presenting with chest pain. The importance of this case highlights not only the reverse subtype of TCM but, more importantly, that stress tests can be a potential contributing factor in this type of cardiomyopathy. Therefore, therapies to limit the extent of myocardial injury need to be researched.
